# The Effect of Acepromazine Alone or in Combination with Methadone, Morphine, or Tramadol on Sedation and Selected Cardiopulmonary Variables in Sheep

**DOI:** 10.1155/2017/7507616

**Published:** 2017-04-05

**Authors:** Lilian Toshiko Nishimura, Isadora Oliveira Junqueira Villela, Leonardo Lamarca Carvalho, Luisa Pucci Bueno Borges, Marcos Augusto Machado Silva, Ewaldo Mattos-Junior

**Affiliations:** ^1^Veterinary Science Graduate Program, University of Franca, Av. Armando Salles Oliveira, No. 201, Pq. Universitário, 14404-600 Franca, SP, Brazil; ^2^The University of Passo Fundo, BR-285, No. 292, Bairro São José, 99010-010 Passo Fundo, RS, Brazil

## Abstract

The sedative and selected cardiopulmonary effects of acepromazine alone or in combination with methadone, morphine, or tramadol were compared in sheep. Six ewes were randomly assigned to treatments: A (0.05 mg/kg acepromazine), AM (A plus 0.5 mg/kg methadone), AMO (A plus 0.5 mg/kg morphine), and AT (A plus 5 mg/kg tramadol). Parameters were assessed before sedative drug administration (baseline) and every 15 minutes thereafter, for two hours. Treatments A and AM were associated with increases in sedation score for 60 minutes and treatments AMO and AT for 30 minutes; however, there were no significant differences between treatments. There was a decrease in mean arterial pressure compared to baseline values in treatment A at 15, 45, 60, and 90 minutes, in treatment AM at 15 minutes, and in treatment AT from 45 to 120 minutes. Arterial blood carbon dioxide pressure increased at all time points in all treatments. Arterial oxygen pressure decreased in treatment AMO at 15, 30, and 120 minutes and in treatment AT at 15–45, 105, and 120 minutes, compared to baseline. Acepromazine alone causes a level of sedation similar to that observed when it is coadministered with opioids methadone, morphine, and tramadol. These combinations did not cause clinical cardiopulmonary changes.

## 1. Introduction

Acepromazine is the most commonly used phenothiazine in veterinary medicine [1]. In ruminants, acepromazine causes sedation and mild skeletal muscle relaxation even in low doses; however, it does not possess antinociceptive properties [1]. Clinical dosing in goats (0.05 mg/kg) has minimal effect on cardiopulmonary function [2]. In dogs, higher intravenous bolus may lead to bradycardia and long-lasting hypotension [3].

A combination of sedatives, such as acepromazine and opioids, is used routinely in dogs in order to potentiate the sedative effects and provide analgesia [4]. Information regarding the effects of such associations in the ovine species in the current literature is sparse. Thus, the purpose of this study was to assess the sedative cardiorespiratory effects of acepromazine alone or in combination with different opioid agents. It was hypothesized that sedation would be superior following the administration of these combinations compared with administration of acepromazine alone.

## 2. Materials and Methods

All procedures involving the use of animals were carried out following approval by the Institutional Animal Welfare Ethics Committee (protocol #038/12).

### 2.1. Animals

Six nonpregnant Santa Inês ewes (mean age, 12 ± 8 months; mean body weight, 39.5 ± 7.4 kg) were used in the study. The animals were confined in 12 m^2^ paddocks, receiving Tifton grass, commercial formulation of ration for sheep, mineral supplementation, and water ad libitum. All animals were clinically healthy upon physical examination, complete blood count, evaluation of renal and hepatic functions, and coproparasitological test. The ewes were acclimated to their surroundings for 20 days prior to the beginning of the study and were accustomed to the presence of the researchers and underwent mild physical restraint daily. Before treatments, the animals were fasted for 24 h and the areas of the right jugular vein and auricular arteries were clipped. The skin in the areas used for vessel catheterization was aseptically prepared. A catheter (18 G) was introduced into the right jugular vein, and a second catheter (20 G) was introduced into an auricular artery with the sheep restrained in a standing position. The study was performed at about 1040 meters above sea level.

### 2.2. Experimental Design

The ewes were randomly assigned (by drawing of lots) to treatments, in a crossover study. All ewes received all treatments, and a seven-day washout period was allowed between treatments. The treatments were as follows: A, acepromazine (0.05 mg/kg, Acepran™ 0.2%, Vetnil, São Paulo, SP, Brazil); AM, A and methadone (0.5 mg/kg, Mytadom™ 10 mg per mL, Cristália, Itapira, SP, Brazil); AMO, A and morphine (0.5 mg/kg, Dimorf™ 10 per mL; Cristália, Itapira, SP, Brazil); and AT, A and tramadol (5 mg/kg, Tramadon™ 50 per mL; Cristália, Itapira, SP, Brazil). Drug combinations were mixed in one syringe and the final volume was adjusted to 5 mL (facilitated blinding), using normal saline solution, and administered via the jugular catheter over 30 seconds.

Data were collected at baseline (time 0, immediately before drug injections) and at 15-minute intervals for 120 minutes following administration of treatments.

### 2.3. Cardiopulmonary Parameters and Rectal Temperature

Heart rate (HR, beats per minute [bpm]) was measured for one minute using a transthoracic stethoscope, on the fifth intercostal space with the point of a flexed elbow used as landmark. Mean arterial pressure (MAP, mmHg) was recorded using the invasive approach, following percutaneous catheterization of the left or right median auricular artery and coupling the catheter connected to a system filled with heparin solution (50 UI/mL) and a calibrated aneroid manometer. The system was zeroed using the air-saline junction at the point of the shoulder in standing and sternally recumbent animals and the xiphoid process in laterally recumbent animals as reference points. Rectal temperature (RT, Celsius degree: °C) was assessed using a transrectal thermometer. Respiratory rate (RR, breaths per minute) was obtained by observation of the thoracic costal movements during a 1-minute period. Arterial blood samples (0.5 mL) were collected from the auricular artery catheter into a heparinized syringe, for assessment of blood pH (pHa), partial pressure of carbon dioxide (PaCO_2_, mmHg), partial pressure of oxygen (PaO_2_, mmHg), bicarbonate (HCO_3_^−^, mmol/L), and base excess (BE, mmol/L). Blood samples were immediately analyzed following sampling using blood pH and gas analyzer (Cobas b121 Roche®, Basel, Switzerland). Corrections were performed based on the values of body temperature. All data were obtained by a single evaluator.

### 2.4. Sedation Assessment

Sedation was assessed using a noninteractive behavioral scale, ranging from 0 to 10 points [5] (Appendix  1, in Supplementary Material available online at https://doi.org/10.1155/2017/7507616), with zero indicating no sedation and 10 maximum sedation. Sedation was scored by three observers who were blinded to the treatments. All of the observers performed the sedation assessment independently and the final score was the arithmetic mean of the three scores.

### 2.5. Statistical Analysis

Data are presented in tables as means, medians, standard deviation, and maximum and minimum values. All parameters were submitted to the Shapiro-Wilk normality test using the software R, followed by analysis using the GraphPad PRISM 5 software. Variables presenting normal distribution were compared among time points using repeated measures one-way ANOVA, followed by Dunnett's posttest; for comparison among treatments, the two-way ANOVA was used, followed by Bonferroni's posttest. Variables presenting nonnormal distribution were compared using Friedman's test, followed by Dunn's posttest for comparison in pairs. Significance level used for all tests was 5%.

## 3. Results

### 3.1. Cardiopulmonary Parameters and Rectal Temperature

Observations on HR, MAP, and RT at time points according to treatment are shown on Table 1. Posttreatment HR did not differ (*P* > 0.05) from baseline values in any of the treatments, and there was no difference (*P* > 0.05) in HR among treatments at any time points.

There was a decrease in MAP in treatment A relative to baseline values at 15 (*P* < 0.05), 45, 60, and 90 minutes (*P* < 0.001), in treatment AMO at 15 minutes (*P* < 0.05), and in AT from 45 to 120 minutes (*P* < 0.001). There was no significant difference (*P* > 0.05) among treatments at any time points.

A decrease (*P* < 0.05) in rectal temperature was observed in animals in treatment A at 45 and 60 minutes and at all time points in AM (*P* < 0.001); however, there was no difference (*P* > 0.05) among treatments.

### 3.2. Arterial Blood Gas Analysis

Results of RR, pHa, PaCO_2_, PaO_2_, HCO_3_^−^, and BD at time points according to treatment are shown on Table 2. Respiratory rate decreased (*P* < 0.01) in comparison to the baseline values at all time points in treatments A and AMO and at 15 and 90 minutes in treatment AM (*P* < 0.05). However, there was no significant difference (*P* > 0.05) among treatments at any time point.

The pHa increased in treatment AMO at 90 minutes (*P* < 0.05) and from 60 to 120 minutes in AT (*P* < 0.001). There was an increase (*P* < 0.01) in PaCO_2_ in all treatments at all time points, but there was no difference (*P* > 0.05) among treatments.

PaO_2_ decreased, in comparison to baseline values, in treatment AMO at 15, 30, and 120 minutes (*P* < 0.05) and at 15, 30, 45, 105, and 120 minutes (*P* < 0.01) in treatment AT. Comparison among treatments revealed a decrease (*P* < 0.05) in PaO_2_ at 15 and 30 minutes in treatments AT and AM.

An increase (*P* < 0.05) was observed in HCO_3_^−^ in comparison to baseline values at all time points in all treatments. Regarding BE, an increase (*P* < 0.05) was seen at 15–120 minutes in treatments A, AM; AMO at 15–105; and at 30–120 minutes in AT, compared to baseline values.

### 3.3. Sedation Score

Signs of excitation of the central nervous system were observed, especially in the animals receiving acepromazine-tramadol, such as sialorrhea, bruxism, vocalization, and increased sensitivity to touch; resolution of these signs occurred within 30 minutes. Animals treatments AM and AMO exhibited the most profound sedation. Two animals in the AM treatment became sternally recumbent (score 6) 30 minutes after premedication and one ewe after 45 minutes. Score 6 was also verified in one animal after treatment AMO at 15 and 60 minutes.

Posttreatment scores differed (*P* < 0.05) from baseline values at 15–60 minutes in animals in treatment A, at 15 (*P* < 0.05), 30–45 (*P* < 0.01), and 60 minutes (*P* < 0.05) in treatment AM, and at 15 (*P* < 0.01) and 30 minutes (*P* < 0.05) in treatments AM and AT (Table 3). There was no significant difference (*P* > 0.05) among treatments at any time point.

## 4. Discussion

To our knowledge, this is the first report using a combination of acepromazine with opioids for sedation in sheep. Because we could not find in the literature equipotent doses of opioids in the ovine species, we chose to use doses described in studies that compared the use of morphine with tramadol or methadone in dogs [6, 7].

Sedative effects of methadone, morphine, and tramadol were widely described in the canine species, and it is known that this association potentiates the sedative effect of acepromazine [4]. It was expected that the association of acepromazine to the opioids could provide better sedation, as described in dogs [4], but the data in the present study suggest that these effects may not occur in sheep. Morphine and fentanyl decrease minimal alveolar concentration of isoflurane in goats and ewes, respectively [8, 9], highlighting the sparing capacity effect of opioids in small ruminants, although these aspects were not assessed in this study.

Opioids usually cause excitation in ruminants [10]. In alpacas and llamas, shivering and signs of nervous system excitation were described following IV administration of tramadol [11, 12]. In sheep, the same signs were observed after injection of 4 and 6 mg/kg of tramadol IV, and the severity of adverse effects was greater in higher doses [13]. No report was found in the literature regarding the effects of morphine or methadone in ewes. However, butorphanol and fentanyl provided behavioral changes such as dysphoria and vocalization [14, 15]. Such behavioral changes could explain the lack of potentiation of sedation of the opioids in combination with acepromazine, compared to that of phenothiazine administered alone. Moreover, due to behavioral peculiarities of the ovine species, interpretation of the effects of sedation is more subjective than in other species. Additionally, we also took into consideration that the low sensitivity of the sedation scoring system used could have hindered the results of this trial.

Significant alteration of HR is more common in doses ≥ 0.1 mg/kg, which occurs in response to hypotension due to blockage of peripheral alfa 1-adrenergic receptors [16]. The results of the present study corroborate the results of a study in goats in which the administration of acepromazine (0.05 mg/kg) intramuscularly had no effect on HR [2].

In this study, the addition of opioid analgesics did not affect HR in ewes. In dogs, negative chronotropism is frequently seen as a result of the increased vagal tone, especially if morphine or methadone is administered [7]. There were few studies using opioids in sheep by the IV route, however, peridural morphine in ewes (0.1 mg/kg) or subcutaneous methadone in goats (0.6 mg/kg) also did not affect HR [17, 18]; nonetheless, this result could be associated with lower plasma concentrations of the drugs following these routes of administration.

The changes in RT associated with the treatments tested in this study were not clinically relevant. Phenothiazines and opioids decrease core temperature as they affect the thermoregulatory center in the central nervous system [1]. This is not usually observed in goats that receive acepromazine alone [2]. In this study, reduction of RT was more frequent in ewes treated with acepromazine-methadone, but baseline values in this treatment were higher compared to those in the other treatments.

Significant changes in pHa occurred at some observation times in the animals that received morphine or tramadol. Increased pHa could explain such rise observed in PaCO_2_. Independently of the cause, increased blood H^+^ without compensation on ventilation is a strong stimuli for the organism to reabsorb HCO_3_^−^, which prevents respiratory acidosis [19], as observed in our trial. However, it was not possible to determinate the cause of those changes on treatments AM and AT, as the animals presented combined metabolic alkalosis (increase in HCO_3_^−^ and, consequently, in BE) and respiratory acidosis.

Acepromazine (0.05 mg/kg, IM) did not affect respiratory and blood gas parameters in goats [2]. In another trial, the same dose of acepromazine was given intramuscularly in cows, leading to increased pHa, HCO_3_^−^, and BE values and decreased PaCO_2_. Such alterations were associated with recumbence and/or hyperventilation [10].

Data on the impact of opioids on blood gas variables in ruminants are sparse in the literature. Butorphanol did not affect blood gas in another study in sheep [14], but potent opioids such as fentanyl can induce transient respiratory depression in this species [15]. Peridural morphine does not lead to respiratory changes in goats [20]; however IV methadone induces hyperventilation [21].

Despite significant changes observed in RR, especially in those animals receiving morphine, it was clear that such changes were not clinically relevant. Furthermore, the reduction of RR reflected minimal changes in PaCO_2_, which was maintained within physiologic range (35–45 mmHg). We highlight that such changes observed in PaCO_2_ could be associated with decreased baseline values, as hyperventilation due to stress of manipulation could have occurred during sampling.

Hypoxemia (PaO_2_ < 60 mmHg) is not usually observed following administration of acepromazine [1]. Although such event did not happen in our study, a decrease in PaO_2_ occurred more markedly in animals given morphine or tramadol. This was concomitant with RR reduction in the animals treated with morphine, which could characterize hypoventilation.

In conclusion, 0.05 mg/kg of acepromazine IV alone provides similar sedation to that produced following its administration in combination with methadone, morphine, or tramadol. Changes in cardiorespiratory parameters were not clinically relevant. However, monitoring PaO_2_ or oxygen peripheral saturation is important, particularly if a combination of acepromazine and morphine or tramadol is being used.

## Supplementary Material

Appendix 1: Numerical rating scale for assessment of sedation in sheep.

## Figures and Tables

**Table 1:** Mean ± standard deviation of heart rate (HR), mean arterial pressure (MAP), and rectal temperature (RT) in six ewes sedated with acepromazine alone (A), acepromazine-methadone (AM), acepromazine-morphine (AMO), or acepromazine-tramadol (AT).

Variable	Treatment	0	15	30	45	60	75	90	105	120
HR beats per minute	A	104 ± 15	105 ± 18	88 ± 8	103 ± 9	99 ± 11	100 ± 13	100 ± 15	100 ± 11	89 ± 13
	AM	100 ± 17	105 ± 18	93 ± 19	98 ± 20	101 ± 21	92 ± 19	101 ± 26	99 ± 30	100 ± 29
	AMO	104 ± 10	109 ± 22	103 ± 23	109 ± 22	107 ± 13	111 ± 26	95 ± 16	100 ± 17	110 ± 20
	AT	100 ± 19	115 ± 28	111 ± 27	101 ± 23	97 ± 27	98 ± 26	92 ± 26	92 ± 23	84 ± 19

MAP (mmHg)	A	114 ± 10	97 ± 11^*∗*^	100 ± 10	98 ± 6^*∗*^	98 ± 8^*∗*^	103 ± 13	93 ± 8^*∗*^	100 ± 9	104 ± 15
	AM	110 ± 9	109 ± 8	105 ± 7	109 ± 11	111 ± 10	109 ± 11	107 ± 9	107 ± 13	108 ± 13
	AMO	105 ± 14	91 ± 5	94 ± 6^*∗*^	98 ± 8	102 ± 6	99 ± 5	100 ± 6	98 ± 9	99 ± 11
	AT	114 ± 10	100 ± 13	100 ± 11	94 ± 11	103 ± 21^*∗*^	94 ± 7^*∗*^	94 ± 7^*∗*^	98 ± 6^*∗*^	91 ± 9^*∗*^

RT °C	A	39.4 ± 0.4	39.1 ± 0.3	39.2 ± 0.3	38.9 ± 0.5^*∗*^	39.0 ± 0.4^*∗*^	39.2 ± 0.2	39.1 ± 0.3	39.1 ± 0.3	39.1 ± 0.5
	AM	39.6 ± 0.6	39.5 ± 0.5^*∗*^	39.3 ± 0.6^*∗*^	39 ± 0.6^*∗*^	39.1 ± 0.5^*∗*^	39.1 ± 0.6^*∗*^	38.9 ± 0.7^*∗*^	39.1 ± 0.4^*∗*^	39.2 ± 0.4^*∗*^
	AMO	39.1 ± 0.5	39.3 ± 0,4	39.2 ± 0.4	39.0 ± 0.5	39.0 ± 0.4	39.0 ± 0.3	39.2 ± 0.2	39.1 ± 0.3	39.3 ± 0.2
	AT	38.8 ± 0.7	39.2 ± 0.7	39.1 ± 0.9	38.8 ± 1.0	38.7 ± 0.9	38.8 ± 1.1	38.8 ± 1.1	38.9 ± 1.0	38.9 ± 1.0

^*∗*^Significantly different from time 0 within the same treatment (*P* < 0.05).

**Table 2:** Mean ± standard deviation of respiratory rate (RR) arterial pH (pHa), partial arterial pressure of carbon dioxide (PaCO_2_), partial arterial pressure of oxygen (PaO_2_), bicarbonate (HCO_3_^−^), and base excess (BD) in six ewes sedated with acepromazine alone (A), acepromazine-methadone (AM), acepromazine-morphine (AMO), or acepromazine-tramadol (AT).

Variable	Treatment	0	15	30	45	60	75	90	105	120
RR breaths per minute	A	28 ± 9	21 ± 6^*∗*^	18 ± 5^*∗*^	17 ± 3^*∗*^	21 ± 4^*∗*^	21 ± 3^*∗*^	21 ± 4^*∗*^	17 ± 2^*∗*^	19 ± 2^*∗*^
	AM	29 ± 9	18 ± 6^*∗*^	25 ± 9	20 ± 3	19 ± 3	22 ± 3	17 ± 3^*∗*^	18 ± 2	21 ± 4
	AMO	28 ± 8	21 ± 8^*∗*^	20 ± 5^*∗*^	17 ± 3^*∗*^	16 ± 4^*∗*^	18 ± 6^*∗*^	18 ± 3^*∗*^	20 ± 4^*∗*^	18 ± 6^*∗*^
	AT	19 ± 4	22 ± 7	17 ± 3	17 ± 2	16 ± 4	18 ± 3	19 ± 3	17 ± 2	17 ± 4

pHa	A	7.51 ± 0.05	7.49 ± 0.03	7.50 ± 0.02	7.51 ± 0.01	7.52 ± 0.03	7.49 ± 0.03	7.50 ± 0.02	7.50 ± 0.01	7.50 ± 0.01
	AM	7.46 ± 0.06	7.48 ± 0.04	7.48 ± 0.03	7.47 ± 0.05	7.47 ± 0.04	7.47 ± 0.03	7.49 ± 0.03	7.49 ± 0.02	7.49 ± 0.02
	AMO	7.46 ± 0.04	7.47 ± 0.02	7.47 ± 0.01	7.47 ± 0.02	7.47 ± 0.03	7.49 ± 0.02	7.53 ± 0.10^*∗*^	7.50 ± 0.03	7.49 ± 0.03
	AT	7.46 ± 0.03	7.46 ± 0.02	7.48 ± 0.03	7.49 ± 0.04	7.49 ± 0.03^*∗*^	7.51 ± 0.04^*∗*^	7.51 ± 0.03^*∗*^	7.52 ± 0.03^*∗*^	7.52 ± 0.02^*∗*^

PaCO_2_ (mmHg)	A	29 ± 2	33 ± 2^*∗*^	34 ± 2^*∗*^	34 ± 2^*∗*^	35 ± 3^*∗*^	34 ± 2^*∗*^	33 ± 2^*∗*^	33 ± 2^*∗*^	33 ± 2^*∗*^
	AM	29 ± 5	33 ± 4^*∗*^	36 ± 4^*∗*^	35 ± 4^*∗*^	35 ± 4^*∗*^	36 ± 4^*∗*^	34 ± 3^*∗*^	35 ± 2^*∗*^	34 ± 4^*∗*^
	AMO	28 ± 2	33 ± 2^*∗*^	34 ± 2^*∗*^	35 ± 1^*∗*^	34 ± 1^*∗*^	33 ± 1^*∗*^	34 ± 2^*∗*^	34 ± 2^*∗*^	34 ± 1^*∗*^
	AT	31 ± 3	34 ± 2^*∗*^	35 ± 2^*∗*^	35 ± 2^*∗*^	36 ± 2^*∗*^	36 ± 3^*∗*^	36 ± 2^*∗*^	36 ± 2^*∗*^	36 ± 2^*∗*^

PaO_2_ (mmHg)	A	80 ± 4	72 ± 3^†^	74 ± 4^†^	75 ± 3	76 ± 4	78 ± 7	72 ± 5	74 ± 3	73 ± 3
	AM	81 ± 1	71 ± 4^†^	72 ± 7^†^	72 ± 6	73 ± 4	73 ± 7	73 ± 2	72 ± 5	74 ± 5
	AMO	81 ± 3	70 ± 5^*∗*†^	73 ± 4^*∗*†^	75 ± 5	72 ± 5	74 ± 6	74 ± 4	78 ± 9	72 ± 5^*∗*^
	AT	82 ± 3	65 ± 12^*∗*†^	68 ± 8^*∗*†^	71 ± 4^*∗*^	69 ± 8	71 ± 8	70 ± 5	70 ± 4^*∗*^	67 ± 6^*∗*^

HCO_3_^−^ (mmol/L)	A	21 ± 3	25 ± 2^*∗*^	26 ± 2^*∗*^	26 ± 2^*∗*^	26 ± 2^*∗*^	26 ± 3^*∗*^	25 ± 2^*∗*^	26 ± 2^*∗*^	25 ± 2^*∗*^
	AM	21 ± 2	24 ± 2^*∗*^	26 ± 2^*∗*^	24 ± 2^*∗*^	25 ± 2^*∗*^	25 ± 3^*∗*^	26 ± 2^*∗*^	26 ± 3^*∗*^	26 ± 2^*∗*^
	AMO	21 ± 1	24 ± 2^*∗*^	25 ± 1^*∗*^	25 ± 2^*∗*^	24 ± 2^*∗*^	25 ± 1^*∗*^	25 ± 2^*∗*^	25 ± 2^*∗*^	25 ± 1^*∗*^
	AT	22 ± 2	24 ± 2^*∗*^	25 ± 2^*∗*^	26 ± 2^*∗*^	27 ± 3^*∗*^	28 ± 3^*∗*^	28 ± 3^*∗*^	29 ± 3^*∗*^	29 ± 3^*∗*^

BE (mmol/L)	A	−1 ± 3	2 ± 3^*∗*^	3 ± 3^*∗*^	3 ± 2^*∗*^	3 ± 2^*∗*^	3 ± 3^*∗*^	2 ± 2^*∗*^	3 ± 2^*∗*^	2 ± 2^*∗*^
	AM	−2 ± 3	1 ± 2^*∗*^	2 ± 2^*∗*^	1 ± 3^*∗*^	1 ± 3^*∗*^	2 ± 2^*∗*^	3 ± 2^*∗*^	3 ± 3^*∗*^	3 ± 1^*∗*^
	AMO	−4 ± 3	0 ± 2^*∗*^	1 ± 1^*∗*^	1 ± 2^*∗*^	1 ± 2^*∗*^	2 ± 1^*∗*^	2 ± 2^*∗*^	2 ± 2^*∗*^	2 ± 2
	AT	−1 ± 2	1 ± 2	2 ± 2^*∗*^	3 ± 3^*∗*^	4 ± 3^*∗*^	5 ± 3^*∗*^	5 ± 3^*∗*^	6 ± 3^*∗*^	5 ± 3^*∗*^

^*∗*^Significantly different from time 0 within the same treatment (*P* < 0.05). ^†^Significantly different from other treatments at the same time point (*P* < 0.05).

**Table 3:** Median (minimum–maximum) of sedation scores in six ewes following intravenous injection of acepromazine alone (A), acepromazine-methadone (AM), acepromazine-morphine (AMO), or acepromazine-tramadol (AT).

Treatment	Time points								
	0	15	30	45	60	75	90	105	120
A	0	1 (1-2)^*∗*^	2 (1-2)^*∗*^	2 (1-2)^*∗*^	1 (1–3)^*∗*^	1 (0–2)	1 (0–2)	1 (0–2)	0 (0-1)
AM	0	3 (1–5)^*∗*^	4 (1–6)^*∗∗*^	3 (1–6)^*∗*^	2 (1–5)^*∗*^	2 (0–6)	2 (0–5)	1 (0-1)	1 (0-1)
AMO	0	3 (1–6)^*∗∗*^	2 (1–4)^*∗*^	4 (0–9)	3 (0–6)	1 (0–2)	1 (0–4)	1 (0–2)	0 (0–2)
AT	0	1 (1–3)^*∗∗*^	1 (1-2)^*∗*^	1 (0–2)	1 (0–2)	0 (0-1)	0 (0-1)	0 (0-1)	0 (0-1)

Statistically different from baseline within treatment ^*∗*^*P* < 0.05; ^*∗∗*^*P* < 0.01. Sedation scores ≥ 6 included sternal recumbency and scores ≥ 8 included lateral recumbency.
