# Supplementary material for: The Effect of Acepromazine Alone or in Combination with Methadone, Morphine, or Tramadol on Sedation and Selected Cardiopulmonary Variables in Sheep
Source: Vet Med Int. 2017 Apr 5;2017:7507616. doi: 10.1155/2017/7507616 (PMC5396443; doi:10.1155/2017/7507616)
Supplement: Supplementary file 1 — Appendix 1: Numerical rating scale for assessment of sedation in sheep. [file 7507616.f1.docx]

**Appendix 1**  Numerical rating scale for assessment of sedation in sheep (Kästner et al. 2003)

| **Score** | **Behavior** |
| --- | --- |
| 0 | Standing, alert, normal behavior |
| 1 | Standing, alert, reduced head and ear movements |
| 2 | Standing, slight head drop |
| 3 | Standing, moderate head drop |
| 4 | Standing, severe head drop and ataxia |
| 5 | Standing, severe head drop and severe ataxia |
| 6 | Sternal recumbency, head up |
| 7 | Sternal recumbency, unable to support head |
| 8 | Lateral recumbency, occasional attempts to attain sternal recumbency |
| 9 | Lateral recumbency, uncoordinated head and leg movements |
| 10 | Lateral recumbency, no movements |
